# Relationship of obesity to physical activity, domestic activities, and sedentary behaviours: cross-sectional findings from a national cohort of over 70,000 Thai adults

**DOI:** 10.1186/1471-2458-11-762

**Published:** 2011-10-04

**Authors:** Emily Banks, Lynette Lim, Sam-Ang Seubsman, Chris Bain, Adrian Sleigh

**Affiliations:** 1 National Centre for Epidemiology and Population Health, Australian National University, Canberra, Australia; 2 Thai Health-Risk Transition Project, School of Human Ecology, Sukhothai Thammathirat Open University, Nonthaburi, Thailand; 3 School of Population Health, University of Queensland, Brisbane, Australia

**Keywords:** Obesity, Thailand, physical activity, inactivity, domestic activity, sedentary behaviours

## Abstract

**Background:**

Patterns of physical activity (PA), domestic activity and sedentary behaviours are changing rapidly in Asia. Little is known about their relationship with obesity in this context. This study investigates in detail the relationship between obesity, physical activity, domestic activity and sedentary behaviours in a Thai population.

**Methods:**

74,981 adult students aged 20-50 from all regions of Thailand attending the Sukhothai Thammathirat Open University in 2005-2006 completed a self-administered questionnaire, including providing appropriate self-reported data on height, weight and PA. We conducted cross-sectional analyses of the relationship between obesity, defined according to Asian criteria (Body Mass Index (BMI) ≥25), and measures of physical activity and sedentary behaviours (exercise-related PA; leisure-related computer use and television watching ("screen-time"); housework and gardening; and sitting-time) adjusted for age, sex, income and education and compared according to a range of personal characteristics.

**Results:**

Overall, 15.6% of participants were obese, with a substantially greater prevalence in men (22.4%) than women (9.9%). Inverse associations between being obese and total weekly sessions of exercise-related PA were observed in men, with a significantly weaker association seen in women (p(interaction) < 0.0001). Increasing obesity with increasing screen-time was seen in all population groups examined; there was an overall 18% (15-21%) increase in obesity with every two hours of additional daily screen-time. There were 33% (26-39%) and 33% (21-43%) reductions in the adjusted risk of being obese in men and women, respectively, reporting housework/gardening daily versus seldom or never. Exercise-related PA, screen-time and housework/gardening each had independent associations with obesity.

**Conclusions:**

Domestic activities and sedentary behaviours are important in relation to obesity in Thailand, independent of exercise-related physical activity. In this setting, programs to prevent and treat obesity through increasing general physical activity need to consider overall energy expenditure and address a wide range of low-intensity high-volume activities in order to be effective.

## Background

The prevalence of obesity is rising rapidly in most Asian countries, with increases of 46% in Japan and over 400% in China observed from the 1980s to early 2000s [1]. In Thailand, the prevalence of obesity increased by around 19% from 1997 to 2004 alone [2]. There have been accompanying increases in morbidity related to conditions such as diabetes and cardiovascular disease in Asian countries [3,4].

It is well established in Western populations that increasing purposeful or leisure-time physical activity (PA) is associated with reduced rates of obesity [5,6]. Recent evidence, also from Western countries, suggests that sedentary activities, such as watching television or using a computer, are associated with increasing obesity, independent of purposeful PA [7-9]. The role of incidental PA and overall energy expenditure, in influencing obesity has been highlighted [10,11]. The interplay between these factors and their combined effects on obesity are not well understood and information relevant to Asian populations is particularly scarce. Furthermore, the relationship between domestic activities and obesity is unclear [12-14]. This is important because physical activity related to patterns of daily activity differs between Asia and Western countries [15] and because many Asian countries are experiencing rapid health and lifestyle transitions [16].

This paper examines in detail the relationships between obesity, exercise-related PA, domestic activities and sedentary behaviours in Thailand, with particular emphasis on the interaction between these factors.

## Methods

### Study population

The Sukhothai Thammathirat Open University (STOU) Cohort Study is designed to provide evidence regarding the transition in risk factor profiles, health outcomes and other factors accompanying development and is described in detail elsewhere [17]. In brief, from April to November 2005, enrolled STOU students across Thailand who had completed a least one semester were mailed a 20-page health questionnaire and asked to join the study by completing the questionnaire, providing signed consent for follow-up, and returning these in a reply-paid envelope. A total of 87 134 men and women aged 15-87 years (median 29 years) joined the cohort.

### Data

All of the variables used in this study were derived from cross-sectional self-reported data from the Thai Cohort Study questionnaire [17]. The questionnaire requested information on: socio-demographic factors; ethnicity; past and present residence and domestic environment; income; work-related factors; height; weight; sensory impairment; mental health; medical history; general health; use of health services; social networks; social capital; diet; physical activity; sedentary behaviours; tobacco and alcohol consumption; use of seat belts and motorcycle helmets; drink-driving; and family structure and health (See Additional files 1 and 2 for questionnaires). Where possible, questionnaire items that had been standardised and validated were used.

Self-reported weight and height were used to calculate participants' BMI, as their weight in kilograms, divided by the square of their height in metres. Cut-points delineating overweight and obesity were set at BMIs ≥23 and ≥25, respectively, in accordance with International Obesity Taskforce recommendations [18] and studies in other Asian populations [19].

Information on exercise-related PA was obtained through a question asking: "During a typical week (7-day period), how many times on average do you do the following kinds of exercise?", with responses requested for: "Strenuous exercise (heart beats rapidly) for more than 20 minutes, e.g. heavy lifting, digging, aerobics or fast bicycling, running, soccer, trakraw"; "Moderate exercise (not exhausting but breathe harder than normal) for more than 20 minutes, e.g. carrying light loads, cycling at a regular pace"; "Mild exercise (minimal effort) for more than 20 minutes, e.g. yoga, Tai-Chi, bowling" and; "Walking for at least 10 minutes e.g. at work, at home, exercise". This question is a sessions-based measure of physical activity, similar to the sessions component of the International Physical Activity Questionnaire and the Active Australia Survey [20]; these session measures have been shown to provide a reliable index of sufficiency of physical activity in non-Thai populations [21]. It incorporates the three major intensities of activity (strenuous, moderate and walking), included in these measures, as well as an additional "mild" category that was created specifically for this study to cover common types of activity in Thailand. The responses to this question were used to derive a weighted measure of overall metabolically-adjusted exercise-related PA, calculated as "2 × strenuous + moderate + mild + walking" exercise sessions, in keeping with previous calculations of this quotient [20].

The frequency of reported housework and gardening was used as a measure of incidental PA and was classified into 5 groups according to the response to the question "How often do you do household cleaning or gardening work?" with options ranging from seldom or never, to most days. Total daily leisure-related screen-time and sitting time were classified according to the participant's response to the question "How many hours per day do you usually spend: Watching TV or playing computer games? Sitting for any purpose (e.g. reading, resting, working thinking)?". Sitting time could also include screen-time, as participants were not specifically asked to exclude screen-time from this measure. The availability of domestic appliances was classified according to the response to the question "Which of the following does your home have now?", with options including microwave oven, refrigerator, water heater and washing machine. The questions on housework/gardening, screen-time, sitting time and availability of domestic appliances were devised specially for the Thai Cohort Study.

Education attainment was classified as: secondary school graduation or less; post-secondary school certificate or diploma; and tertiary graduate. Personal monthly income was in Thai Baht in four categories (≤7,000; 7,001-10,000; 10,001-20,000; >20,000). Respondents recorded the frequency of eating deep fried food and soft drinks and Western-style fast foods such as pizza (known as "junkfood") on a five-point Likert scale ranging from never or less than once a month, to once or more a day; this was categorised as consumption <3 times and ≥ 3 times per week for fried food and seldom (never or less than once per month) and regularly (≥ once per month) for "junkfood". Fruit and vegetable intakes were noted as serves eaten per day and categorised as <2 and ≥2 serves per day. They were asked if they have ever smoked, when they started and when they quit and were categorised as current smokers or not current smokers, with similar questions and categories for alcohol consumption.

Analysis was restricted to the 95% of individuals aged between 20 and 50 years, with BMIs between 11 and 50. Individuals were excluded from the analyses if they were missing data on age or sex (n = 2), height or weight (n = 1030) or physical activity or inactivity (n = 6863), leaving 74,981 participants.

### Statistical methods

The relationships between a range of personal characteristics and exercise-related PA, housework/gardening and leisure-related screen-time were examined, as well as the correlation between the individual measures of physical activity and inactivity. Variables were categorised into the groups listed in the various tables.

The proportion of the study population classified as obese according to exercise-related PA, housework, leisure-related screen-time and sitting time was examined. Prevalence odds ratios (OR) and 95% CIs for obesity according to PA, housework, screen-time and sitting time were estimated using unconditional logistic regression; crude and adjusted odds ratios were computed. ORs were presented separately for men and women and adjusted for age (as a continuous variable), income and educational attainment, with exploration of the effect of additional adjustment for factors such as marital status, smoking, alcohol consumption and urban/rural residence. We evaluated the significance of interaction terms using a likelihood ratio test, comparing the model with and without the interaction terms.

We examined how much of any association of a specific PA or sedentary behaviour with obesity was attributable to differences in total physical activity level by modelling simultaneously the three PA variables and their two-way and three-way interactions. We also examined how much of the association of certain sedentary behaviours could be attributed to the effect of other sedentary behaviours and to consumption of fried foods and soft drinks and Western-style junkfood, using mutual adjustment.

All analyses were carried out in STATA version 9.2. All statistical tests were two-sided, using a significance level of p < 0.05. Due to the large sample size, conclusions were based on both significance and the effect size.

### Ethical approval

Ethics approval was obtained from Sukhothai Thammathirat Open University Research and Development Institute (protocol 0522/10) and the Australian National University Human Research Ethics Committee (protocol 2004344). Informed written consent was obtained from all participants.

## Results

Of 74 981 participants with appropriate data, 41 351 (55.2%, 95%CI 54.8-55.5%) were classified as being of healthy weight (BMI 18.5-22.9), 10 733 (14.4%, 14.1-14.6%) were underweight (BMI < 18.5), 11 241 (15.0%, 14.7-15.2%) were overweight but not obese (BMI ≥ 23.0-24.9) and 11 616 (15.6%, 15.2-15.7%) were obese (BMI ≥ 25.0).

Men were far more likely to be overweight (21.7%, 21.3-22.1%) or obese (22.4%, 22.0-22.9%) than women (9.5% and 9.9%, respectively), while women were more likely to be underweight (21.3%, 20.9-21.7%) than men (5.9%, 5.6-6.1%). Compared to other members of the study cohort, obesity prevalence was higher in older participants and urban dwellers and in those with higher consumption of fried food (data not shown) [22].

Patterns of exercise-related PA varied between men and women, with 12.5% (12.2-12.9%) of men reporting 0-3 sessions and 26.3% (25.8-26.8%) reporting ≥18 sessions of exercise-related PA per week compared to 22.2% (21.8-22.6%) and 12.1% (11.8-12.4%), respectively, for women. The mean number of sessions of exercise-related PA per week was 11.6 [sd 12.1] overall; 13.9 [sd 13.5] for men and 9.7 [sd 10.6] for women. A higher level of exercise-related PA was associated with having less than a tertiary education, being of lower income and eating more fruit and vegetables, but was not strongly related to other factors (Table 1). The pattern of PA making up the total weekly sessions also differed between the sexes, with women much less likely than men to report strenuous or moderate PA (Table 2).

**Table 1:** Characteristics of study population according to total physical activity, housework/gardening and daily screen-time

	total physical activity (per week)		housework/gardening (per week)		leisure screen-time (per day)		Total
	**<7 sessions**	≥**7 sessions**	**<2 times**	≥**2 times**	**<3 hours**	≥**3 hours**	

TOTAL (n)	31995	42986	33373	41608	35936	39045	74981
							
male (% [n])	33.3 [10668]	53.3 [22928]	52.2 [17413]	38.9 [16178]	48.5 [17455]	41.3 [16136]	44.8 [33591]
age (mean [SD], years)	30.2 [7.2]	30.2 [7.4]	30.1 [7.2]	30.2 [7.3]	31.3 [7.5]	29.1 [6.9]	30.2 [7.3]
married (%[n])	44.1 [13792]	43.0 [17983]	40.2 [13117]	46.2 [18658]	50.0 [17472]	37.5 [14303]	43.5 [31775]
urban resident (% [n])	55.5 [17656]	49.0 [20958]	57.7 [19143]	47.1 [19471]	48.6 [17359]	54.8 [21255]	51.8 [38614]
tertiary educated (% [n])	27.9 [8917]	23.4 [10029]	30.3 [10091]	21.3 [8855]	24.5 [8778]	26.1 [10168]	25.3 [18946]
income ≥10,000 Baht per month (% [n])	36.8 [11539]	33.7 [14150]	40.9 [13437]	30.2 [12252]	38.7 [13645]	31.6 [12044]	35.0 [25689]
limited physical function^a ^(%[n])	10.0 [3185]	9.2 [3912]	9.3 [3073]	9.7 [4024]	9.6 [3432]	9.4 [3665]	9.5 [7097]
current smoker, males only^b ^(% [n])	22.0 [2325]	21.1 [4766]	21.9 [3768]	20.9 [3323]	20.7 [3556]	22.2 [3535]	21.4 [7091]
regular alcohol drinker, males only^b ^(% [n])	12.4 [1314]	9.3 [2109]	11.7 [2035]	8.6 [1388]	9.6 [1670]	10.9 [1753]	10.2 [3423]
eat fried food daily (% [n])	15.9 [5065]	15.0 [6401]	15.0 [4977]	15.7 [6489]	14.0 [4994]	16.7 [6472]	15.4 [11466]
consume soft drinks/junkfood (% [n])	29.1 [9326]	29.3 [12555]	30.6 [10188]	28.2 [11693]	26.4 [9451]	31.9 [12430]	29.2 [21881]
vegetable intake (mean [SD], serves/day)	1.7 [1.3]	2.1 [1.7]	1.8 [1.5]	2.0 [1.6]	1.8 [1.5]	2.0 [1.6]	1.9 [1.6]
fruit intake (mean [SD], serves/day)	2.4 [2.1]	3.0 [2.6]	2.5 [2.2]	3.0 [2.5]	2.6 [2.3]	2.9 [2.5]	2.8 [2.4]
							
physical activity (mean [SD], sessions/week)	3.9 [2.5]	17.3 [13.3]	10.1 [11.0]	12.8 [12.9]	11.8 [12.4]	11.4 [11.9]	11.6 [12.1]
housework ≥2 times/week (% [n])	48.6[15535]	60.7 [26073]			56.3 [20235]	54.7 [21373]	55.5 [41608]
sleeping (mean [SD], hours/day)	6.9 [2.2]	6.9 [2.2]	7.0 [2.1]	6.9 [2.3]	6.7 [2.3]	7.1 [2.2]	6.9 [2.2]
leisure screen-time (mean [SD], hours/day)	3.0 [1.9]	2.9 [1.8]	3.0 [1.9]	2.9 [1.9]	1.5 [0.6]	4.2 [1.7]	2.9 [1.9]
sitting (mean [SD], hours/day)	6.8 [3.9]	6.4 [3.8]	6.7 [3.9]	6.4 [3.8]	6.1 [3.9]	7.0 [3.7]	6.6 [3.8]
							
body-mass index (mean [SD], kg/m2)	21.6 [3.6]	21.9 [3.3]	22.0 [3.5]	21.6 [3.3]	21.9 [3.3]	21.7 [3.6]	21.8 [3.4]
							
Participants with missing values are not included in the percentages							

^a^Measured as top decile from 3 items assessing physical limitations in the past 4 weeks (eg how much bodily pain did you have in the past 4 weeks?)

^b^Only 1% and 0.6%, respectively, of females are current smokers and regular drinkers

**Table 2:** Relationship between being obese and measures of exercise-related physical activity (PA)

	MALES				FEMALES			
	**Total** **n**	**Obese** **n (%)**	**age adjusted** **OR (95% CI)**	**adjusted** OR (95% CI)*	**Total** **n**	**Obese** **n (%)**	**age adjusted** **OR (95% CI)**	**adjusted** OR (95% CI)*

**strenuous exercise-related physical activity >20 mins**								
0 sessions/week	10924	2772 (25.4)	1.00	1.00	23972	2341 (9.8)	1.00	1.00
1 sessions/week	4518	1073 (23.8)	0.96 (0.89-1.05)	0.96 (0.88-1.04)	5774	543 (9.4)	1.03 (0.93-1.13)	1.04 (0.94-1.15)
2 sessions/week	4214	945 (22.4)	0.90 (0.82-0.98)	0.90 (0.83-0.99)	4271	451 (10.6)	1.14 (1.02-1.27)	1.11 (1.00-1.24)
3 sessions/week	4409	938 (21.3)	0.83 (0.76-0.90)	0.83 (0.76-0.90)	3721	353 (9.5)	0.92 (0.82-1.04)	0.91 (0.81-1.03)
4 sessions/week	2390	517 (21.6)	0.85 (0.77-0.95)	0.86 (0.77-0.96)	1081	131 (12.1)	1.20 (0.99-1.45)	1.22 (1.01-1.48)
5+ sessions/week	7136	1281 (18.0)	0.70 (0.65-0.75)	0.71 (0.66-0.77)	2571	271 (10.5)	0.99 (0.87-1.14)	0.99 (0.86-1.13)
p(trend)				<0.0001				0.98
								
**moderate exercise-related physical activity >20 mins**								
0 sessions/week	12171	2991 (24.6)	1.00	1.00	23459	2321 (9.9)	1.00	1.00
1 sessions/week	4297	1003 (23.3)	0.97 (0.89-1.05)	0.95 (0.87-1.04)	5677	550 (9.7)	1.06 (0.96-1.17)	1.07 (0.97-1.18)
2 sessions/week	4677	1022 (21.9)	0.89 (0.82-0.97)	0.88 (0.81-0.96)	4647	429 (9.2)	0.98 (0.88-1.09)	0.97 (0.87-1.09)
3 sessions/week	4066	859 (21.1)	0.85 (0.78-0.93)	0.84 (0.77-0.92)	3333	316 (9.5)	0.96 (0.85-1.09)	0.97 (0.87-1.10)
4 sessions/week	1826	375 (20.5)	0.81 (0.71-0.91)	0.81 (0.72-0.92)	1081	124 (11.5)	1.17 (0.96-1.42)	1.15 (0.94-1.40)
5+ sessions/week	6554	1276 (19.5)	0.79 (0.73-0.85)	0.81 (0.75-0.87)	3193	350 (11.0)	1.13 (1.00-1.27)	1.10 (0.98-1.25)
p(trend)				<0.0001				0.19
								
**mild exercise-related physical activity >20 mins**								
0 sessions/week	20590	4671 (22.7)	1.00	1.00	27176	2838 (10.4)	1.00	1.00
1 sessions/week	3482	815 (23.4)	1.04 (0.95-1.14)	1.03 (0.94-1.12)	4598	419 (9.1)	0.90 (0.81-1.01)	0.93 (0.83-1.04)
2 sessions/week	2388	509 (21.3)	0.90 (0.81-1.00)	0.90 (0.81-1.00)	3219	298 (9.3)	0.91 (0.79-1.02)	0.92 (0.81-1.05)
3 sessions/week	1879	423 (22.5)	0.96 (0.85-1.07)	0.96 (0.85-1.08)	2486	210 (8.5)	0.77 (0.66-0.90)	0.78 (0.67-0.91)
4 sessions/week	939	237 (25.2)	1.10 (0.94-1.28)	1.10 (0.94-1.29)	815	65 (8.0)	0.75 (0.58-0.97)	0.72 (0.56-0.94)
5+ sessions/week	4313	871 (20.2)	0.84 (0.78-0.92)	0.87 (0.80-0.94)	3096	260 (8.4)	0.74 (0.65-0.85)	0.73 (0.64-0.84)
p(trend)				0.004				<0.0001
								
**walking for **≥**10 minutes**								
0 sessions/week	6588	1488 (22.6)	1.00	1.00	6528	726 (11.1)	1.00	1.00
1 sessions/week	2696	698 (25.9)	1.17 (1.05-1.30)	1.17 (1.05-1.30)	3538	391 (11.1)	1.03 (0.90-1.18)	1.03 (0.90-1.18)
2 sessions/week	2345	560 (23.9)	1.10 (0.94-1.18)	1.06 (0.94-1.19)	3484	375 (10.8)	1.03 (0.90-1.17)	1.02 (0.89-1.17)
3 sessions/week	2636	658 (25.0)	1.11 (1.00-1.24)	1.12 (1.00-1.25)	4194	392 (9.4)	0.87 (0.76-0.99)	0.86 (0.75-0.98)
4 sessions/week	1559	378 (24.3)	1.09 (0.96-1.25)	1.11 (0.97-1.27)	2039	211 (10.4)	1.01 (0.86-1.20)	1.00 (0.83-1.16)
5+ sessions/week	17767	3744 (21.1)	0.94 (0.88-1.01)	0.98 (0.91-1.05)	21607	1995 (9.2)	0.87 (0.80-0.95)	0.84 (0.77-0.92)
p(trend)				0.06				<0.0001
								
**weighted total sessions exercise-related physical activity**								
0-3 sessions/week	4201	1180 (28.1)	1.00	1.00	9199	1022 (11.1)	1.00	1.00
4-6 sessions/week	4199	1032 (24.6)	0.85 (0.77-0.94)	0.85 (0.77-0.94)	7639	744 (9.7)	0.90 (0.81-1.00)	0.90 (0.81-0.99)
7-11 sessions/week	8640	1997 (23.1)	0.81 (0.75-0.89)	0.82 (0.75-0.90)	12775	1190 (9.3)	0.85 (0.78-0.93)	0.84 (0.77-0.92)
12-17 sessions/week	7722	1645 (21.3)	0.74 (0.68-0.81)	0.76 (0.70-0.83)	6783	612 (9.0)	0.81 (0.73-0.90)	0.79 (0.71-0.88)
18+ sessions/week	8829	1672 (18.9)	0.66 (0.60-0.72)	0.69 (0.63-0.75)	4994	525 (10.5)	0.95 (0.85-1.07)	0.92 (0.82-1.03)
p(trend)				<0.0001				0.002

*adjusted for age, income and education

Overall, 49.4% (48.9-49.9%) of women and 34.4% (33.8-34.8%) of men reported doing household cleaning or gardening on most days of the week, while 3.7% (3.5-3.9%) of women and 8.8% (8.5-9.1%) of men reported that they did these seldom or never. Housework/gardening was more common among those who were married, not tertiary educated, of lower income and with greater fruit and vegetable intake than other cohort members (Table 1).

Leisure related screen-time did not vary markedly between men and women. 17.8% (17.4-18.2%) of women and 22.2% (21.8-22.7%) of men reported less than two hours of daily screen-time, while 3.4% (3.2-3.6%) of women and 2.8% (2.6-2.9%) of men reported 8 hours or more. Average daily leisure related screen-time was 2.9 hours [sd 1.9]; it was 3.0 hours [sd 1.9] in women and 2.8 hours [sd 1.8] in men. Higher levels of screen-time were more common among cohort members who were younger, unmarried, urban residents and of lower income and who ate fried food daily and soft drinks or Western style junkfood once a month or more often (Table 1).

Women tended to have greater levels of sitting time than men, with 46.6% (46.0-46.9%) of women and 36.8% (36.2-37.3%) of men reporting 8 or more hours of daily sitting time. Average daily sitting time was 6.6 hours [sd 3.8] overall; 6.8 hours [sd 3.9] in women and 6.2 hours [sd 1.8] in men.

The number of hours of daily screen-time was poorly but significantly inversely correlated with the number of weekly sessions of exercise-related PA (r = -0.016; 95% CI: -0.024 to -0.009) and doing household cleaning or gardening (r = -0.022; -0.029 to -0.014) but was more strongly and positively related to number of hours sitting per day (r = 0.16; 0.15 to 0.16). The number of weekly sessions of exercise-related PA was positively correlated with doing household cleaning or gardening (r = 0.15; 0.14 to 0.16). The correlations between sitting time and number of weekly sessions of exercise-related PA and cleaning/gardening were -0.054 (-0.061 to -0.047) and -0.041 (-0.049 to -0.034) respectively.

### Obesity and exercise-related physical activity, housework, and gardening

In men, the OR for being obese decreased steadily and significantly with increasing weighted total weekly sessions of exercise-related PA, such that those reporting 18 or more sessions had a OR of obesity of 0.69 (0.63-0.75) compared to those with 0-3 sessions (p(trend) < 0.0001) and this relationship was observed particularly for moderate and strenuous PA (Table 2). There was no apparent relationship between being obese and moderate and strenuous PA in women and the relationship between strenuous activity and obesity differed significantly between men and women (p(interaction) < 0.0001). However, in women an inverse relationship with being obese was observed mainly for mild PA and walking (Table 2).

**Figure 1:**
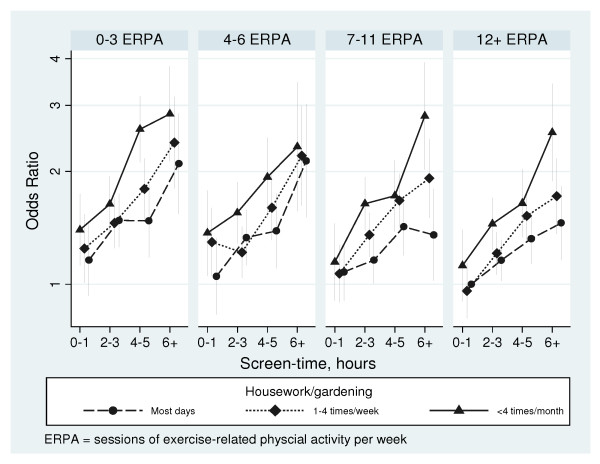
**Odds ratios (OR) for being obese in relation to weighted weekly sessions of exercise-related physical activity (ERPA), hours of daily screen-time and frequency of housework/gardening**.

For both sexes, the risk of being obese was consistently lower with increasing frequency of housework/gardening, with 33% (26-39%) and 33% (21-43%) lower adjusted ORs in men and women, respectively, in those reporting these activities daily versus seldom or never (Table 3). The lower risk of obesity with increasing housework or gardening was independent of the level of exercise-related PA, in that the OR did not change materially (i.e. changed by <10%) following additional adjustment for exercise-related PA (see below) and a similar relationship was observed within separate categories of exercise-related PA (Figure 1). The inverse relationship between housework/gardening and obesity was still present following additional adjustment for screen-time and exercise-related PA. Compared to people who did housework or gardening seldom or never, the ORs (95% CI) for being obese in men were: 0.85 (0.76-0.94) for people who did housework or gardening 1-3 times per month; 0.79 (0.71-0.87) for 1-2 times per week; 0.80 (0.72-0.90) for 3-4 times per week; and 0.73 (0.66-0.80) for housework/gardening on most days, adjusting for age, income, education, screen-time and weighted weekly sessions of exercise-related PA. For women, the ORs for the same categories were: 0.95 (0.79-1.15); 0.76 (0.64-0.90); 0.75 (0.63-0.90); and 0.71 (0.60-0.84), respectively.

**Table 3:** Relationship between being obese and gardening/housework, leisure-related computer or television use ("screen-time") and sitting time

	MALES				FEMALES			
	**Total** **n**	**Obese** **n (%)**	**age adjusted** **OR (95% CI)**	**adjusted** OR (95% CI)*	**Total** **n**	**Obese** **n (%)**	**age adjusted** **OR (95% CI)**	**adjusted** OR (95% CI)*

**gardening or housework**								
seldom or never	2962	806 (27.2)	1.00	1.00	1550	198 (12.8)	1.00	1.00
1-3 times/month	4960	1217 (24.5)	0.85 (0.77-0.95)	0.83 (0.75-0.93)	3200	378 (11.8)	0.96 (0.80-1.16)	0.94 (0.78-1.13)
1-2 times/week	9491	2092 (22.0)	0.78 (0.71-0.86)	0.76 (0.69-0.84)	11210	1066 (9.5)	0.76 (0.64-0.89)	0.74 (0.62-0.87)
3-4 times/week	4688	1040 (22.2)	0.76 (0.68-0.85)	0.76 (0.68-0.85)	5076	482 (9.5)	0.77 (0.64-0.92)	0.72 (0.60-0.87)
Most days	11490	2371 (20.6)	0.65 (0.59-0.72)	0.67 (0.61-0.74)	20354	1966 (9.7)	0.73 (0.63-0.86)	0.67 (0.57-0.79)
p(trend)				<0.0001				<0.0001
								
**screen-time**								
0-1 hours/day	7452	1568 (21.0)	1.00	1.00	7339	673 (9.2)	1.00	1.00
2-3 hours/day	17109	3862 (22.6)	1.23 (1.15-1.32)	1.21 (1.13-1.29)	20694	1935 (9.4)	1.15 (1.04-1.26)	1.15 (1.04-1.26)
4-5 hours/day	6875	1560 (22.7)	1.42 (1.31-1.54)	1.38 (1.27-1.50)	10112	1089 (10.8)	1.49 (1.35-1.65)	1.51 (1.36-1.67)
6-7 hours/day	1227	300 (24.5)	1.61 (1.39-1.87)	1.58 (1.36-1.84)	1847	202 (10.9)	1.57 (1.32-1.86)	1.63 (1.38-1.94)
8+ hours/day	928	236 (25.4)	1.85 (1.57-2.18)	1.85 (1.56-2.18)	1398	191 (13.7)	2.18 (1.83-2.60)	2.16 (1.81-2.59)
p(trend)				<0.0001				<0.0001
								
**sitting-time**								
0-1 hours/day	2115	510 (24.1)	1.00	1.00	2600	250 (9.6)	1.00	1.00
2-3 hours/day	7651	1661 (21.7)	0.88 (0.79-0.99)	0.88 (0.79-1.00)	7797	701 (9.0)	0.97 (0.83-1.13)	0.95 (0.81-1.11)
4-5 hours/day	7102	1598 (22.5)	0.92 (0.82-1.04)	0.92 (0.82-1.04)	6850	714 (10.4)	1.14 (0.98-1.33)	1.14 (0.98-1.33)
6-7 hours/day	4248	928 (21.8)	0.87 (0.77-0.99)	0.86 (0.76-0.98)	4709	464 (9.9)	1.00 (0.85-1.18)	1.02 (0.84-1.20)
8+ hours/day	12296	2792 (22.7)	0.95 (0.85-1.06)	0.92 (0.82-1.03)	19177	1933 (10.1)	1.11 (0.96-1.283)	1.14 (0.99-1.31)
p(trend)				0.85				0.001

*adjusted for age, income and education

numbers do not always sum to total due to missing values

### Obesity and leisure related screen-time

Increasing leisure-related screen-time was associated with significantly and substantially increasing risk of being obese in both men and women, with 85% and 116% increases in risk, respectively, for 8 or more hours of daily screen-time versus <2 hours (Table 3, p(trend) < 0.0001). Overall, sitting-time was not significantly related to the OR of being obese (p(trend) = 0.32), although a significant trend was observed in women (Table 3).

The positive relationship between screen-time and being obese was still present following additional adjustment for housework/gardening and exercise-related PA; the ORs (95% CI) for obesity in men were: 1.22 (1.14-1.30); 1.38 (1.27-1.50); 1.58 (1.36-1.83); and 1.80 (1.53-2.13) and for women were: 1.15 (1.05-1.27); 1.50 (1.35-1.67); 1.62 (1.36-1.92) and 2.13 (1.78-2.55), for people with: 2.0-2.9 hours; 3.0-3.9 hours; 4-7.9 hours; and ≥8 hours of daily screen-time versus 0-1.9 hours, respectively, adjusted for age, income, education, housework/gardening and exercise-related PA. Additional adjustment for consumption of fried foods, soft drink and junkfood and smoking and alcohol consumption did not materially alter the OR (data not shown).

Figure 1 shows the cohort divided into four groups according to their level of exercise-related PA (ERPA in the figure). The ORs for being obese were then presented within each group according to the number of hours of total daily screen-time, with separate lines according to the frequency of housework/gardening. This figure shows increasing risk of being obese with increasing screen-time within each exercise-related PA group and within each housework/gardening group. It also shows that the finding of lower risks of being obese with increasing frequency of housework/gardening persists, even when screen-time and exercise-related PA were accounted for. When the relationships between being obese and exercise-related PA, screen-time and housework/gardening were modelled together, no significant interactions were observed (likelihood ratio χ_39_^2 ^= 38.54, p = 0.49), indicating that they were each independently associated with obesity.

**Figure 2:**
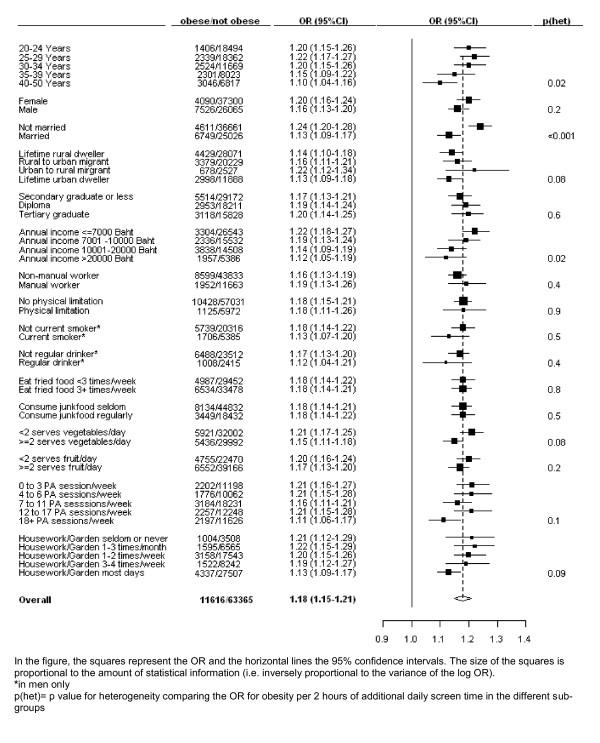
**Odds ratios (OR) for being obese per 2 hour increase in daily screen-time, in different population sub-groups, adjusted for age, sex, income and education, where appropriate**.

The sex, income and education-adjusted OR of obesity per two-hour increase in daily screen-time is shown separately according to a variety of factors, including according to total exercise-related PA and according to housework and gardening, in Figure 2. There was an 18% (15-21%) increase in the risk of being obese with every two additional hours of daily screen-time overall and a significant elevation in the risk of being obese with increasing screen-time was seen in all of the population sub-groups examined (Figure 2). There was a significantly greater increase in the risk of being obese with increasing screen-time in unmarried compared to married individuals (p(heterogeneity) < 0.0001). The relationship between being obese and screen-time was attenuated significantly in older cohort members (p(heterogeneity) = 0.02) and in those with higher incomes (p(heterogeneity) = 0.02). No significant variation in the relationship between screen-time and being obese was seen according to the other factors examined, including: sex; urban/rural residence history; education; smoking status; alcohol, fruit, vegetable, junkfood and fried food intake; disability; level of exercise-related PA; and frequency of housework/gardening.

### Obesity and domestic appliances

The risk of being obese was significantly higher in men and women from households with a refrigerator, microwave oven or washing machine and in men from households with a water heater (Table 4). The risk of being obese increased significantly with the increasing number of such appliances within a household. These results were not altered materially following additional adjustment for housework/gardening frequency, smoking, alcohol consumption and consumption of fried foods, Western-style junkfood, fruit and vegetables (data not shown).

**Table 4:** Relationship between being obese and ownership of household appliances

	MALES				FEMALES			
	**Total** **n**	**obese** **n (%)**	**age adjusted** **OR (95% CI)**	**adjusted** OR (95% CI)*	**Total** **n**	**obese** **n (%)**	**age adjusted** **OR (95% CI)**	**adjusted** OR (95% CI)*

**washing machine**								
no	10414	1499 (14.4)	1.00	1.00	12071	845 (7.0)	1.00	1.00
yes	23368	6080 (26.0)	1.68 (1.58-1.79)	1.57 (1.47-1.68)	29849	3296 (11.0)	1.31 (1.21-1.43)	1.36 (1.25-1.47)
								
**refrigerator**								
no	2256	276 (12.2)	1.00	1.00	1901	118 (6.2)	1.00	1.00
yes	31526	7303 (23.2)	1.70 (1.49-1.94)	1.53 (1.34-1.75)	40019	4023 (10.1)	1.29 (1.07-1.57)	1.32 (1.09-1.61)
								
**microwave oven**								
no	24182	4749 (19.6)	1.00	1.00	28139	2482 (8.8)	1.00	1.00
yes	9600	2830 (29.5)	1.43 (1.35-1.51)	1.34 (1.26-1.42)	13781	1659 (12.0)	1.11 (1.04-1.19)	1.17 (1.09-1.26)
								
**water heater**								
no	24269	4810 (19.8)	1.00	1.00	29129	2623 (9.0)	1.00	1.00
yes	9513	2769 (29.1)	1.33 (1.26-1.41)	1.24 (1.17-1.32)	12791	1518 (11.9)	1.02 (0.95-1.09)	1.07 (0.99-1.15)
								
**number of appliances**								
0	1973	222 (11.3)	1.00	1.00	1546	88 (5.7)	1.00	1.00
1	7487	1065 (14.2)	1.23 (1.05-1.44)	1.20 (1.02-1.40)	8916	585 (6.6)	1.09 (0.96-1.23)	1.08 (0.86-1.37)
2	11865	2676 (22.6)	1.89 (1.63-2.19)	1.76 (1.51-2.05)	14515	1458 (10.0)	1.56 (1.38-1.76)	1.53 (1.22-1.92)
3	7038	1899 (27.0)	2.13 (1.83-2.48)	1.94 (1.66-2.27)	9278	1045 (11.3)	1.60 (1.41-1.81)	1.55 (1.23-1.96)
4	5419	1717 (31.7)	2.49 (2.13-2.90)	2.22 (1.89-2.61)	7665	965 (12.6)	1.66 (1.46-1.88)	1.58 (1.25-2.00)
								

*adjusted for age, income and education

## Discussion

In this cohort of Thai men and women, the risk of being obese is consistently higher in those with greater time spent in leisure-related television watching and computer games and inversely associated with time spent doing housework or gardening. Inverse associations between obesity and total weekly sessions of exercise-related PA are observed in men with a significantly weaker association seen in women. Exercise-related PA, screen-time and housework/gardening each have independent associations with obesity. The magnitude of the association with obesity relating to these risk factors is substantial. Individuals reporting daily housework/gardening have a 33% lower risk of being obese compared to those reporting these activities seldom or never and there is an 18% increase in the risk of obesity with every two hours of additional daily screen-time.

The findings reported here show an inverse relationship between exercise-related PA and being obese that is stronger in men than in women and may be somewhat weaker than that observed in Western populations. The inverse relationship between obesity and exercise, usually leisure-related PA is well established in Western countries [5,6,21]. Although a reduced risk of obesity with increasing PA has been demonstrated in certain Asian populations, including those in China [23] and Korea [24], the specific relationship of leisure-related PA to obesity is less clear, and may be of lesser magnitude. The reason for this is not known. Potential explanations include: the lack of data relevant to Asia; the possibility that the proportion of total energy expenditure to leisure-related PA is lower in the Asian context [15]; differing types and intensities of leisure-related PA compared to the West; and differences in measurement error.

We were unable to locate any previous studies in adults of the relationship between being obese and television and computer use in Asia. Studies in Western populations consistently show increases in obesity with increasing time spent in sedentary activities, particularly screen-time [7,8,25-27]. The direct relationship between sedentary behaviours and obesity is observed in both cross-sectional [7,25,26] and prospective studies [8,15,28]. Studies have varied in the way they have measured and categorised screen-time and other sedentary behaviours, as well as obesity related outcomes, so it is difficult to summarise quantitatively the magnitude of the risk involved. However, the 18% increase in obesity risk per 2 hours of additional daily screen-time observed here is consistent with the 23% increase observed in US nurses [8] and older Australian adults [9].

The one previous publication we were able to locate examining the relationship between BMI and domestic activity in the Asian context demonstrated a significantly lower BMI in men with increasing time spent in domestic activities and a non-significant relationship in women, in China [29]. Studies in Western populations have generally not found a significant relationship between BMI and domestic activity [12,13,30], even heavy domestic activity, although one study in older US adults found house cleaning, but not gardening, to be associated with decreased BMI on multivariate analysis [14] and another found decreased all-cause mortality with increasing domestic PA [30]. The study presented here is the largest to date investigating the issue and shows a decreasing risk of being obese with increasing frequency of housework and gardening, independent of exercise-related PA and screen-time.

Although the lack of a positive finding in the Western context may reflect measurement error, the play of chance, small sample sizes or other factors, it is also possible that domestic PA in Asian countries differs from that in Western countries, for example, due to use of labour saving devices or differing practices. Increasing use of labour saving devices is part of the transition accompanying industrialisation and is associated with reduced energy expenditure in domestic tasks [31]. Decreasing domestic physical activity over time has been noted in one Chinese study [29]. We found household ownership of domestic appliances to be significantly associated with increasing risk of being obese, with increasing risks of being obese accompanying increasing numbers of appliances within the household. However, the lack of specificity in the relationship of the different household appliances to obesity and the apparently greater effect in men compared to women suggests that this may well not be a causal relationship; it may instead reflect a broader difference in socioeconomic status and lifestyle between households with and without appliances.

Strengths of the current study include its large size and inclusion of adults from a wide range of social and economic backgrounds. Although the cohort is somewhat younger and more urbanised than the Thai general population, it represents well the geographic regions of Thailand and exhibits substantial heterogeneity in the distribution of other factors [17]. For example, 35% of males and 47% of females had low incomes (<7000 Baht per month or $5.50 US per day). Participants in the Thai Cohort Study in 2005 were very similar to the STOU student body in that year for sex ratio, age distribution, geographic region of residence, income, education and course of study [32]. Much of the health-risk transition underway in middle income countries is mediated by education [33,34] and the cohort is, by definition, ahead of national education trends. It is therefore likely to provide useful early insights into the effects and mediators of the health-risk transition in middle-income countries. Previous relevant studies from the cohort include examination of the broader health-related correlates of obesity [22] and the relationship between gender, socioeconomic status and obesity [35].

The proportion of the cohort classified as overweight but not obese is similar to the 18% found in the third Thai National Health Examination Survey (2004), while obesity is much lower among STOU women (10% compared to 36% in the National Health Examination Survey) but similar among men in the two studies (23%) [2]. It should be noted that the Thai Cohort Study, like the vast majority of cohort studies, is not designed to be representative of the general population, but is meant to provide sufficient heterogeneity of exposure to allow reliable estimates of relative risk based on internal comparisons [36]. The "healthy cohort effect" and the 44% response rate for this study means that the estimates of relative risk shown here are likely to be conservative, since community members with more extreme behaviours and health conditions may be less likely to be attending an open university or to participate. However, it is important to note that ORs comparing groups *within *the cohort remain valid and can be generalised more broadly [36,37]. Furthermore, the major comparisons in this paper are between obese and non-obese individuals, rather than obese and "healthy weight" individuals, which is also likely to lead to more conservative estimates of association.

The limitations of the study should also be borne in mind. The measures used for tobacco smoking and alcohol consumption were brief and the physical activity measure used has, to our knowledge, only been validated in Western populations. BMI was based on self-reported height and weight, which have been shown to provide a valid measure of body size in this population, with correlation coefficients for BMI based on self-reported versus measured height and weight of 0.91 for men and 0.95 for women [38]. However, BMI based on self-reported measures was underestimated by an average of 0.77 kg/m^2 ^for men and 0.62 kg/m^2 ^for women [38]. Cut-points delineating overweight and obesity were set at BMIs ≥23 and ≥25; weight-related disease in Asian populations occurs least when BMI is about 22 or less [39], and significantly increases with BMI ≥23 [40]. The excellent correlation between measured and self-reported values mean that self-reported values are generally reliable for ranking participants according to BMI in epidemiological studies, as was done here. The general underestimation of BMI means that absolute values of BMI from self-reported measures are less reliable.

Self-reported leisure-related television and computer use has good test-retest reliability and validity, although respondents have a tendency to under-report the number of hours involved [41]. We were not able to locate any studies validating these measures in Asian populations. Time spent in habitual, incidental physical activity is difficult to measure [42] and domestic activities, screen-time and overall physical activity are all likely to be reported with differing degrees of measurement error. Although both domestic activities and screen-time remain predictive of obesity within categories of physical activity, it is possible that this is the result of greater measurement error in ascertaining overall physical activity than in ascertaining domestic and screen-related activities [25]. However, the lack of strong correlation between screen-time and overall physical activity (r = -0.016) goes against this argument, as does the previous observation that television viewing remains a significant predictor of obesity in women, following adjustment for pedometer measured physical activity [42].

Obesity and overweight are the result of sustained positive energy balance, whereby energy intake exceeds energy expenditure. Although dietary factors are important, there is mounting evidence that insufficient energy expenditure is likely to be a key factor underlying the global obesity epidemic [10]. The main determinant of individual energy expenditure is the basal metabolic rate, which typically accounts for 70% of all kilojoules burned [15]. A further 10% of energy expenditure comes from the thermic effect of food and the remaining 20% comes from PA [15]. PA is often conceptualised as comprised of purposeful and non-purposeful physical activity; the latter is also termed "incidental" PA. A recent study from the US found that over half of population-level energy expenditure from PA was from sedentary and low-intensity tasks, 16% was attributed to occupational activity above and beyond sitting at work, 16% was attributable to domestic activities and yard work of at least moderate intensity and less than 5% was attributable to leisure-time PA [43]. This evidence is consistent with the suggestion that differences in incidental physical activity are responsible for the greatest variations in energy expenditure between individuals and populations [10]. Moreover, recent evidence indicates that one of the most potent mechanisms determining cardiovascular risk factors, including obesity and metabolic disorders, is the amount of time spent in high volume daily intermittent low-intensity postural or ambulatory activities, which account for as much of 90% of energy expended in physical activity [11].

Sedentary behaviours generally involve sitting or lying down and are characterised by low energy expenditure (metabolic equivalent intensity <2) [41]. A substantial amount of time spent in sedentary activities is likely to contribute to obesity through reduced overall energy expenditure, mainly resulting from their impact on incidental physical activity, since it may co-exist with relatively high levels of exercise-related physical activity. Screen-time, particularly television watching, is also associated with other health behaviours, such as eating fatty foods. However, the finding of increased obesity among those watching greater amounts of television persisted in this dataset after adjustment for intake of fatty food and in other studies following adjustment for total energy intake [8] and foods eaten while watching television [27], so this is unlikely to explain much of its effects.

In lower- and middle-income countries, including Thailand, industrialisation is generally accompanied by increasing urbanisation, a more sedentary lifestyle, with increasing car and computer use and a higher fat diet dominated by more refined foods [16]. It is also characterised by a shift in work patterns for a substantial proportion of the population, from high energy expenditure activities such as farming, mining and forestry to less energy-demanding jobs in the service sector [16]. All of these changes are likely to increase population obesity. There are a number of specific barriers to increasing physical activity in many Asian countries, including environmental factors such as heat, inadequate urban infrastructure, pollution and other hazards. Furthermore, chronic malnutrition has been common in many Asian countries, leading to stunting in significant portions of the population and rendering them vulnerable to obesity as food availability improves.

The importance of obesity, the metabolic syndrome and diabetes in Thailand has been highlighted extensively [44-46]. In Thailand, and in many other countries, social factors are key upstream determinants of the major influences on obesity. For example, domestic duties are often divided along gender lines and many wealthier households have servants, particularly to do the heavier work. In this cohort, higher socioeconomic status was accompanied by increasing risk of being obese in men and decreasing risk of being obese in women [35]. This pattern is believed to represent an intermediate stage in the health-risk transition between less-developed countries such as China, which demonstrate high socioeconomic status to be associated with increased obesity in both men and women [47], and Western populations where high socioeconomic status is associated with reduced obesity in both men and women [2,35].

The study was able to investigate simultaneously a number of activity-related measures and the large numbers allowed quantification of the association of these factors with obesity within a range of population subgroups. However, the analyses presented here are cross-sectional so it is not possible to directly attribute causality to the relationships observed or to exclude reverse causality. Reverse causality occurs when an exposure varies because of the specific condition under investigation. In this case, reverse causality would mean that obesity might result in reduced exercise-related PA, increased sedentary behaviour and decreased domestic PA. There are *a priori *reasons why it is likely that certain elements of the PA-BMI relationship are causal i.e. that reduced energy expenditure due to reduced PA results in increased BMI. However, it is also possible that people with high BMI may change their level of PA. Intuitively, this might apply more to exercise-related PA than to screen-time or domestic activities; people with a high BMI may do more exercise-related PA in order to lose weight or may reduce their exercise-related PA, due to the extra exertion required because of their weight or obesity related health issues (e.g. joint problems). In Thai society, women in particular are under pressure to be thin and the increased walking among women of higher BMI may reflect this. Going against a large role for reverse causality is the fact that increasing inactivity has been shown to result in increased obesity in longitudinal data [8,48] and experimental studies show that increasing BMI by overfeeding of lean individuals does not result in increased sedentary behaviour [49]. This issue is not resolved entirely by using longitudinal data, since the major risk factor for incident obesity is having a high BMI at baseline [49]. We propose that the relationship between sedentary behaviour and obesity is likely to be complex, with a causal relationship between inactivity and obesity predominating. There is likely to be some contribution of obesity leading to inactivity [48], or indeed a "spiral" relationship, whereby inactivity leads to obesity, which further exacerbates inactivity, leading to further increases in obesity [50].

## Conclusions

In common with many middle to low income countries, the prevalence of overweight and obesity in Thailand is lower than that seen in many Western nations, but is increasing rapidly. Avoiding the transition to the obesity patterns seen in the West is a key priority. The data presented here suggest that habitual, high volume, low intensity PA is likely to be important for maintaining a healthy weight and are in keeping with other data that show that increasing exercise-related leisure-time PA alone is unlikely to be sufficient to prevent population obesity [15]. Leisure-related television and computer use were strongly related to the risk of being obese. Research focusing on habitual activities and sedentary behaviours is relatively new. Effective interventions to reduce sedentary time and increase incidental activity are being developed; innovative interventions applicable to the Asian context are needed urgently.

## Competing interests

The authors declare that they have no competing interests.

## Authors' contributions

EB, LL, CB, AS and SS contributed to the study concept and design. EB and LL wrote the initial draft of the manuscript. LL performed the statistical analysis. All authors were involved in the interpretation of the analyses and critically revising the manuscript for important intellectual content. AS and SS provided study supervision and were involved in the acquisition of data. All authors read and approved the final manuscript.

## Pre-publication history

The pre-publication history for this paper can be accessed here:

http://www.biomedcentral.com/1471-2458/11/762/prepub

## Supplementary Material

Additional file 1 **Thai Cohort Study baseline questionnaire (English)**. An English language translation of a questionnaire administered to students of Sukhothai Thammathirat Open University in 2005. Click here for file

Additional file 2 **Thai Cohort Study baseline questionnaire (Thai)**. The Thai language original questionnaire administered to students of Sukhothai Thammathirat Open University in 2005. Click here for file
